# [Retracted] Downregulation of CKS1B restrains the proliferation, migration, invasion and angiogenesis of retinoblastoma cells through the MEK/ERK signaling pathway

**DOI:** 10.3892/ijmm.2024.5382

**Published:** 2024-05-13

**Authors:** Zhou Zeng, Zhao-Lin Gao, Zhi-Pei Zhang, Hai-Bo Jiang, Chang-Quan Yang, Jie Yang, Xiao-Bo Xia

Int J Mol Med 44: 103-114, 2019; DOI: 10.3892/ijmm.2019.4183

Following the publication of the above paper, it has been drawn to the Editors' attention by a concerned reader that certain of the lumen formation assay data shown in Fig. 5A on p. 112 were strikingly similar to data appearing in different form in another article written by different authors at different research institute, which had already been published in the journal *Biomedicine & Pharmacotherapy* prior to the submission of this paper to *International Journal of Molecular Medicine*, and which has also subsequently been retracted. In view of the fact that the contentious data had already apparently been published previously, the Editor of *International Journal of Molecular Medicine* has decided that this paper should be retracted from the Journal. After having been in contact with the authors, they agreed with the decision to retract the paper. The Editor apologizes to the readership for any inconvenience caused.

